# Clinical outcomes and mortality before and after implementation of a pediatric sepsis protocol in a limited resource setting: A retrospective cohort study in Bangladesh

**DOI:** 10.1371/journal.pone.0181160

**Published:** 2017-07-28

**Authors:** Teresa Bleakly Kortz, David M. Axelrod, Mohammod J. Chisti, Saraswati Kache

**Affiliations:** 1 Department of Pediatrics, University of California, San Francisco School of Medicine, San Francisco, California, United States of America; 2 Department of Pediatrics, Stanford School of Medicine, Stanford, California, United States of America; 3 Nutrition and Clinical Services Division, International Centre for Diarrhoeal Disease Research, Bangladesh, Dhaka, Bangladesh; National Yang-Ming University, TAIWAN

## Abstract

**Background:**

Pediatric sepsis has a high mortality rate in limited resource settings. Sepsis protocols have been shown to be a cost-effective strategy to improve morbidity and mortality in a variety of populations and settings. At Dhaka Hospital in Bangladesh, mortality from pediatric sepsis in high-risk children previously approached 60%, which prompted the implementation of an evidenced-based protocol in 2010. The clinical effectiveness of this protocol had not been measured. We hypothesized that implementation of a pediatric sepsis protocol improved clinical outcomes, including reducing mortality and length of hospital stay.

**Materials and methods:**

This was a retrospective cohort study of children 1–59 months old with a diagnosis of sepsis, severe sepsis or septic shock admitted to Dhaka Hospital from 10/25/2009-10/25/2011. The primary outcome was inpatient mortality pre- and post-protocol implementation. Secondary outcomes included fluid overload, heart failure, respiratory insufficiency, length of hospital stay, and protocol compliance, as measured by antibiotic and fluid bolus administration within 60 minutes of hospital presentation.

**Results:**

404 patients were identified by a key-word search of the electronic medical record; 328 patients with a primary diagnosis of sepsis, severe sepsis, or septic shock were included (143 pre- and185 post-protocol) in the analysis. Pre- and post-protocol mortality were similar and not statistically significant (32.17% vs. 34.59%, p = 0.72). The adjusted odds ratio (AOR) for post-protocol mortality was 1.55 (95% CI, 0.88–2.71). The odds for developing fluid overload were significantly higher post-protocol (AOR 3.45, 95% CI, 2.04–5.85), as were the odds of developing heart failure (AOR 4.52, 95% CI, 1.43–14.29) and having a longer median length of stay (AOR 1.81, 95% CI 1.10–2.96). There was no statistically significant difference in respiratory insufficiency (pre- 65.7% vs. post- 70.3%, p = 0.4) or antibiotic administration between the cohorts (pre- 16.08% vs. post- 12.43%, p = 0.42).

**Conclusions:**

Implementation of a pediatric sepsis protocol did not improve all-cause mortality or length of stay and may have been associated with increased fluid overload and heart failure during the study period in a large, non-governmental hospital in Bangladesh. Similar rates of early antibiotic administration may indicate poor protocol compliance. Though evidenced-based protocols are a potential cost-effective strategy to improve outcomes, future studies should focus on optimal implementation of context-relevant sepsis protocols in limited resource settings.

## Introduction

Of the 5.9 million global deaths in children under age 5 in 2015[1], the majority resulted from sepsis: the final common pathway for most infectious disease-related deaths.[2–4] Sepsis and septic shock are secondary to a systemic inflammatory response, and represent a clinical spectrum of a dysregulated host response when exposed to an infection that can result in severe, multi-organ dysfunction,[5–7] including cardiovascular (CV) dysfunction, and frequently Acute Respiratory Distress Syndrome (ARDS).[8] Clinically, this manifests as a febrile and lethargic child, with oliguria, tachycardia, and inadequate tissue perfusion. Pediatric sepsis is a serious, life-threatening condition with high morbidity and mortality,[2–4, 8–13] with an estimated global prevalence of 8.2% amongst children in the intensive care unit (ICU) and an in-hospital mortality rate of 25% worldwide.[14]

The bulk of sepsis mortality occurs in limited resource settings, where HIV, malaria, tuberculosis, malnutrition, limited access to care, and late presentation frequently complicate management. Sepsis is both preventable and treatable; prompt recognition and early, appropriate therapy in the initial hours can greatly impact survival,[15–19] while delays in presentation and/or diagnosis are known risk factors for poor outcomes in limited resource settings.[17, 19]

In 2011, Maitland *et al*., published results from a large, multicenter, randomized controlled trial: Fluid Expansion as Supportive Therapy (FEAST).[12] In this trial, African children with severe febrile illness who received recommended aggressive fluid resuscitation had a significantly higher relative risk (RR) of mortality compared to the control group (RR for any fluid bolus vs. control, 1.45; 95% CI, 1.13 to 1.86; P = 0.003).[12] Current international guidelines recommend fluid resuscitation for septic shock,[8, 16, 20–23] but the controversial results from the FEAST trial call into question whether current fluid resuscitation guidelines, primarily developed in resource-rich settings, may be causing more harm than good in limited resource settings. Still, protocol-driven approaches to sepsis management have been shown to not only improve patient outcomes in resource-rich settings,[23–29] but do so in a cost-effective manner.[25] In this study, we evaluated if implementaion of a sepsis management protocol in a large, non-governmental hospital in Bangladesh had similar benefit. We also evaluated the impact of standard, aggressive fluid resusciation on outcomes in pediatric patients in this hospital.

Dhaka Hospital, operated by the International Centre for Diarrhoeal Disease Research, Bangladesh (icddr,b), delivers medical care to approximately 140,000 patients per year with primarily diarrheal and acute respiratory illnesses, with or without associated complications or comorbid conditions.[30] Dhaka Hospital and its staff care for thousands of children, hundreds with sepsis, every year. Mortality amongst children who demonstrate signs of systemic inflammation and have documented bacteremia approaches 33%[31] and mortality ranges from 14% to 67% in those with severe sepsis and septic shock respectively.[32]

**Fig 1:**
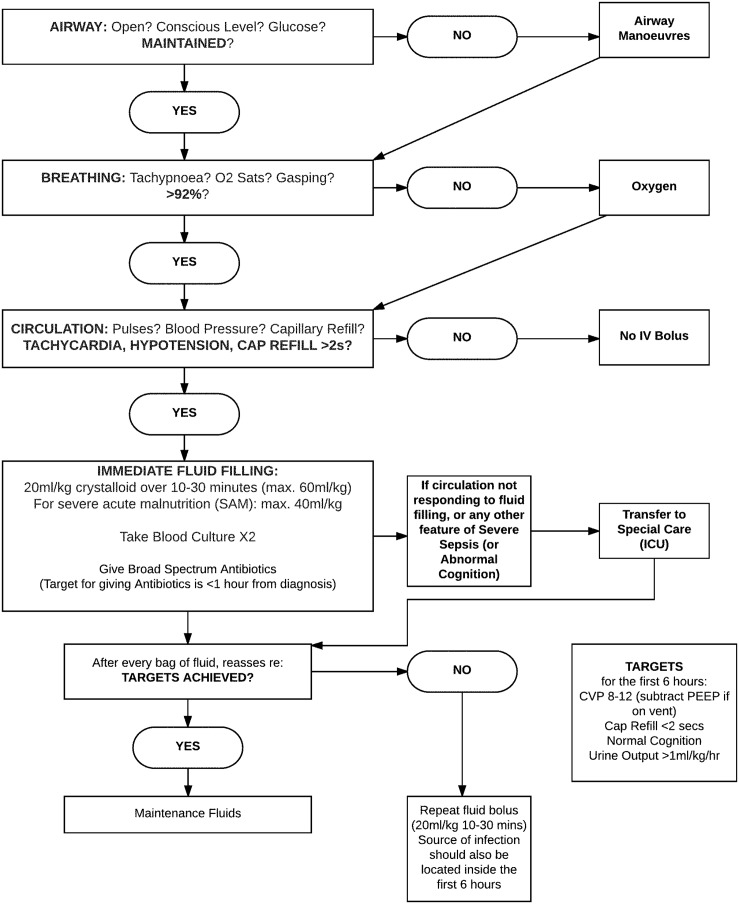
icddr,b Pediatric sepsis protocol adapted from the Surviving Sepsis Campaign.

In 1999, Dhaka Hospital adopted a protocolized approach to the management of severe, acute malnutrition (SAM), which included an algorithm for septic shock in severely malnourished children.[33] Implementation of a hospital-wide protocol is a relatively easy, cost-effective strategy that can improve morbidity and mortality, offering an ideal intervention in limited resource settings.[25] In the Dhaka Hospital ICU, approximately two thirds of the patients who develop septic shock have concomitant SAM;[31] after implementation of the 1999 SAM treatment protocol, mortality decreased from 17% to 8.5% in this population,[33] offering evidence that implementation of a pediatric sepsis protocol could be successful in this setting. Subsequently, in October 2010, Dhaka Hospital implemented a Pediatric Sepsis Protocol (Fig 1) developed using the Surviving Sepsis Campaign International Guidelines to improve recognition and treatment of sepsis.[8, 34] Our retrospective cohort study explores the effectiveness of this evidenced-based pediatric sepsis protocol. Given prior success with protocol implementation in this and other settings, we hypothesized that implementation of a pediatric sepsis protocol would improve clinical outcomes, including reducing mortality and length of hospital stay.

## Materials and methods

### Study design and population

This retrospective cohort study was conducted at Dhaka Hospital using the electronic medical record (EMR) database, named SHEBA, which captures demographic data, laboratory results, physician and nursing documentation, vital signs, microbiologic results, and medication administration data. The study population was children ages 1–59 months admitted with sepsis, severe sepsis, or septic shock according to the International Pediatric Sepsis Consensus Definitions[8] (Box 1) as a primary diagnosis one year before through one year after a pediatric sepsis protocol was implemented: 10/25/09-10/25/11. We identified subjects with a keyword search (“sepsis”, “septic”) of the EMR.

**Box 1. Definitions**

**Under weight**[35]: Determined by weight-for-age z-score (WAZ); composite of acute and chronic malnutrition.

**Sepsis**[8]: Infection (documented or suspected) AND ≥2 abnormalities, one of which must be temperature or white blood cell count (WBC): temperature >38.5°C or <35°C, heart rate >2 SD above normal or bradycardia if age <1 year, respiratory rate >2 SD above normal for age, elevated/decreased white blood cell count for age or >10% bands, altered mental status.

**Severe sepsis**[8]: Sepsis AND cardiovascular dysfunction (hypotension, capillary refill >2sec despite adequate fluid) OR Acute Respiratory Distress Syndrome (ARDS; S_p_O_2_ <92% in room air) OR ≥2 organ dysfunctions (altered mental status, decreased GCS by ≥3, platelet count <80,000 or INR >2, creatinine ≥2 times normal, increased bilirubin >4mg/dl or ALT ≥2 times normal.

**Septic shock**[8]: Severe sepsis AND cardiovascular dysfunction despite fluid resuscitation requiring vasoactive infusion to maintain blood pressure in the normal range.

**Sclerema**[36]: Diffuse, doughy feeling of the skin and/or hardening of the subcutaneous tissue in the absence of any localized skin lesion often associated with a severe, underlying infection.

The primary outcome was change in inpatient mortality rate after implementation of the Pediatric Sepsis Protocol. Secondary clinical outcomes evaluated included changes in rates of fluid overload, heart failure, and respiratory insufficiency, as well as length of hospital stay. Fluid overload was determined by the presence of any two of the following phrases during a keyword search of physician notes: fluid overload, ascites, bilateral crackles, rales, pulmonary edema, tachypnea, gallop (without hepatomegaly), peripheral edema, and hepatomegaly. Heart failure was defined as the presence of any one of the following phrases: heart failure, cardiac failure, poor cardiac output, gallop with hepatomegaly, or congestive heart failure. Respiratory insufficiency was defined as the presence of any one of the following phrases: mechanical ventilation, intubation, oxygen, continuous positive airway pressure (CPAP), bubble CPAP, nasal cannula, or ventilator. Length of hospital stay was determined from the EMR. We collected data on severity of illness using the Pediatric Index of Mortality 2 Score (PIM2), which calculates a predicated death rate at the time of the first face-to-face contact. The PIM2 was chosen because it relies less on laboratory values than other scores, as results are rarely available in real-time in this setting (S1 Table).[37]

For all patients included in the analysis, a single researcher (TBK) performed all chart reviews to determine if the patient met criteria for sepsis, severe sepsis, or septic shock (Box 1). We also extracted data on vital signs, nutritional status (Box 1), mental status, time to antibiotic administration, time to crystalloid fluid bolus administration, volume of crystalloid received, whole blood volume received, and laboratory results. Sepsis protocol compliance was determined by antibiotic and fluid bolus administration within one hour of admission. This study was approved by Stanford University, Stanford, CA, USA (protocol ID 26167, IRB Number 4947, Panel 6, approved 03/08/2013), and the Research and Ethical Review Committees at the icddr,b in Dhaka, Bangladesh (protocol number 12102, approved 04/13/2013).

### Sample size and selection

**Fig 2:**
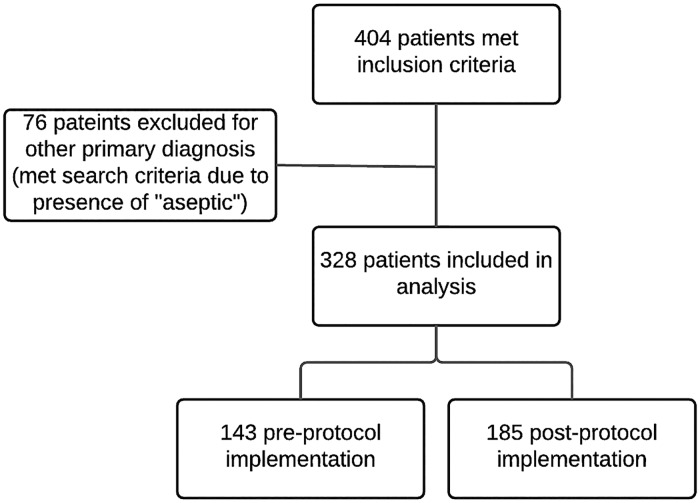
Subject identification and inclusion in the analysis.

Many significant changes occurred at Dhaka Hospital in the years shortly after protocol implementation, including general staff and physician training in intensive care management and resuscitation, as well as upgrades in the ICU monitoring and equipment. Given the timing of these changes and of protocol implementation, the research team decided to limit the study period to one year before and one year after implementation to limit confounding due to the other known factors. In that period of time, 404 patient charts were identified by a keyword search for “sepsis” or “septic”, 76 of which were excluded upon chart review for having a primary diagnosis other than sepsis, severe sepsis or septic shock (“aseptic” triggered the key word search) (Fig 2). 328 subjects were identified as meeting inclusion criteria: 143 pre- and 185 post-protocol. A post-hoc sensitivity analysis (G*Power 3.1) given the actual sample size of 328, a two-tailed α of 0.05, and 80% power would have detected a difference in mortality of ≥14%.

### Statistical analysis

Data analyses for mortality and clinical outcomes used a combination of R 3.0.1 and STATA 14.2, and the following tests: t-test (means), Fisher’s exact and Kruskal-Wallis tests (contingency tables), binomial test (comparing individual factor levels), Wilcoxon rank-sum test (medians), and logistic regression analyses (odds ratios). We performed unadjusted and adjusted logistic regression analyses for mortality, length of stay, and secondary clinical outcomes. We adjusted for *a priori* selected predictor variables present on admission and key interventions to explore the potential effect of treatment on outcome: age in months, sex, weight-for-age Z-score (WAZ), PIM2 score, hematocrit, meets sepsis criteria, culture positive sepsis, antibiotic administration within 60 minutes, protocol status, crystalloid volume received (none, 1–20, 21–60, and >60 ml/kg), and blood volume received (none, 1–10, >11 ml/kg). Strata for crystalloid volume were based on the recommended fluid bolus volume per the Pediatric Sepsis Protocol, which was 20 ml/kg to be repeated up to a maximum volume of 60 ml/kg, and strata for whole blood volume were based on the WHO recommendations for septic shock (10ml/kg), which was the clinical practice at Dhaka Hospital. We tested for interaction not only between nutritional status (WAZ score) and fluid bolus volume, but also nutritional status and blood volume, and between fluid bolus and whole blood transfusion. No interactions were detected, and the variables were treated as independent in the logistic regression. A probability <0.05 was considered statistically significant and strength of association was determined by odds ratios and 95% confidence intervals. Data were de-identified prior to analysis.

## Results

### Patient characteristics

328 patients were included in the analysis. The two cohorts were comparable in regards to the majority of the measured baseline patient characteristics (Table 1), with the exception of the median predicted probability of death based on the PIM2 Score (2.8% pre- vs. 3.7% post-protocol, p = 0.01) and the percentage of children that met septic shock criteria (16.1% pre- vs. 33.0% post-protocol, p = 0.001).

**Table 1:** Baseline patient characteristics.

Characteristic	Pre-protocol (N = 143)	Post-protocol (N = 185)	p-value
Mean age in months (SD)	9.5 (11.3)	8.9 (10.0)	0.59
Male, n (%)	79 (55.2)	108 (58.4)	0.58
Median predicted probability of death based on PIM2 score, % (IQR)	2.8 (2.5–3.1)	3.7 (3.2–4.2)	0.01
Hypoxia at admission, n (%)	94 (65.7)	130 (70.3)	0.40
Bacteremia, n (%)	39 (27.3)	53 (28.7)	0.81
Hematocrit, mean % (SD)	30.1 (7)	31.6 (7)	0.06
Sepsis severity			
Sepsis, n (%)	131 (91.6)	171 (92.4)	0.78
Severe sepsis, n (%)	95 (66.4)	136 (73.5)	0.16
Septic shock, n (%)	23 (16.1)	61 (33.0)	0.001
Malnutrition			
Any degree of malnutrition (WAZ <-1 SD), n (%)	129 (90.2)	171 (92.4)	0.32
Severe malnutrition (WAZ <-3 SD), n (%)	85 (59.4)	123 (66.5)	0.32

SD: Standard deviation; PIM2: Pediatric Index of Mortality 2 Score; IQR: Interquartile range; WAZ: Weight-for-age Z-score

### Mortality

**Fig 3:**
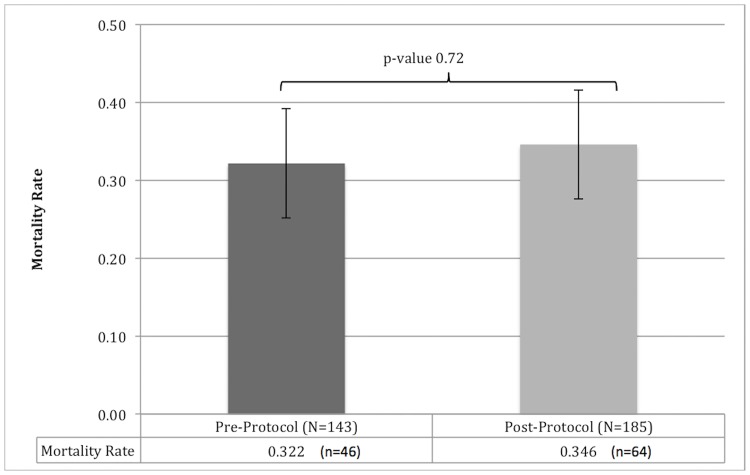
Mortality rate pre- and post-protocol implementation. Error bars represent 95% confidence intervals and the p-value for mortality pre- and post-protocol implementation is shown.

Pre- and post-protocol mortality (Fig 3) were similar and not statistically significant (32.2% vs. 34.6%, p = 0.72). For patients who died in each cohort, we calculated the median total volume (crystalloid plus blood) and median volume of each fluid type (crystalloid or blood) received (Table 2). The median total and crystalloid bolus volume were similar between the two cohorts and not statistically significant (41 vs. 47 ml/kg, p = 0.28, and 37 vs. 36 ml/kg, p = 0.76, pre- and post-protocol, respectively). Those that died in the post-protocol cohort received more blood than the pre-protocol cohort, which was statistically significant (0 vs. 4 ml/kg, p = 0.002, pre- and post-protocol, respectively).

**Table 2:** Mortality pre- and post-protocol by fluid type (crystalloid vs. blood) received.

Volume received	Pre-protocol (N = 42) ^†^	Post-protocol (N = 63) ^†^	p-value
Total volume (crystalloid + blood), median (IQR) (ml/kg)	41 (11–68)	47 (11–69)	0.28
Crystalloid bolus, median (IQR) (ml/kg)	37 (10–63)	36 (20–52)	0.76
Whole blood (ml/kg), median (IQR) (ml/kg)	0 (0–0)	4 (0–16)	0.002

^†^ Limited to subjects with available bolus and blood volume data.

IQR: Interquartile range.

### Secondary clinical outcomes

We analyzed differences in clinical outcomes pre- and post-protocol implementation, including fluid overload, heart failure, respiratory insufficiency, and median length of hospital stay. There were statistically significant increased rates of fluid overload (31.5 vs. 54.1%, p-value <0.001) and heart failure (3.5 vs. 10.3%, p-value = 0.02), as well as a longer median length of hospital stay (96hrs vs. 120hrs, p = 0.02) in the post-protocol cohort (Table 3). There was no statistically significant difference between rates of respiratory insufficiency between cohorts (65.7 vs. 70.3%, p = 0.4, respectively) (Table 3).

**Table 3:** Secondary clinical outcomes.

Outcome	Pre-Protocol (N = 143)	Post-Protocol (N = 185)	p-value
**Fluid overload, n (%)** [95% CI]	45 (31.5) [23.8–39.1]	100 (54.1) [46.9–61.3]	<0.001
**Heart failure, n (%)** [95% CI]	5 (3.5) [0.5–6.5]	19 (10.3) [5.9–14.7]	0.02
**Respiratory insufficiency, n (%)** [95% CI]	94 (65.7) [57.9–73.5]	130 (70.3) [63.7–76.9]	0.40
**Median length of stay (hours)** (IQR)	96 (48–168)	120 (72–240)	0.02

95% CI: 95% confidence interval; IQR: Interquartile range.

### Logistic regression

A logistic regression for mortality post-protocol (Table 4) showed an unadjusted odds ratio (OR) of 1.12 (95% CI, 0.70–1.77). We performed a multivariable logistic regression, which resulted an adjusted OR (AOR) of 1.55 (95% CI, 0.88–2.71). We considered the effect of antibiotic administration within 60 minutes on mortality, which resulted an AOR of 1.01 (95% CI 0.47–2.22). Reduced logistic regression models including statistically and clinically relevant predictors were also performed and yielded similar results.

**Table 4:** Unadjusted and adjusted ORs for mortality and secondary clinical outcomes stratified by volume and type fluid (crystalloid and blood).

	Unadjusted OR	95% CI	p-value	Adjusted OR	95% CI	p-value
Mortality						
Post-Protocol	1.12	0.70–1.77	0.64	1.55^†^	0.88–2.71	0.13
Antibiotics within 60 min	2.37	1.47–3.82	<0.001	1.01^Ŧ^	0.47–2.22	0.96
Crystalloid Bolus Volume (ml/kg)						
None	1	-	-	1	-	-
1–20	3.52	1.54–8.03	0.003	2.72^Ŧ^	0.84–8.81	0.10
21–60	4.75	2.46–9.16	<0.001	2.90^Ŧ^	1.01–8.31	0.05
>60	3.96	1.83–8.53	<0.001	2.50^Ŧ^	0.80–7.81	0.12
Whole Blood Volume (ml/kg)						
None	1	-	-	1	-	-
1–10	4.53	1.80–11.42	0.001	2.65^Ŧ^	0.86–8.16	0.09
>11	3.39	1.83–6.30	<0.001	1.84^Ŧ^	0.80–4.27	0.15
Fluid Overload						
Post-protocol	2.56	1.62–4.04	<0.001	3.45^†^	2.04–5.85	<0.001
Crystalloid Bolus Volume (ml/kg)						
None	1	-	-	1	-	-
1–20	1.36	0.66–2.82	0.41	0.74^Ŧ^	0.26–2.12	0.58
21–60	1.99	1.16–3.42	0.01	0.78^Ŧ^	0.30–1.98	0.59
>60	2.11	1.08–4.10	0.03	0.81^Ŧ^	0.29–2.25	0.69
Whole Blood Volume (ml/kg)						
None	1	-	-	1	-	-
1–10	4.68	1.75–12.50	0.002	2.61^Ŧ^	0.89–7.71	0.08
>11	8.74	0.41–0.69	<0.001	5.71^Ŧ^	2.19–14.90	<0.001
Heart Failure						
Post-protocol	3.16	1.15–8.68	0.03	4.52^†^	1.43–14.29	0.01
Crystalloid Bolus Volume (ml/kg)						
None	1	-	-	1	-	-
1–20	3.73	1.21–11.52	0.02	2.48^Ŧ^	0.37–16.73	0.35
21–60	0.73	0.22–2.46	0.61	0.38^Ŧ^	0.05–2.92	0.35
>60	1.63	0.48–5.61	0.44	0.82^Ŧ^	0.11–6.24	0.85
Whole Blood Volume (ml/kg)						
None	1	-	-	1	-	-
1–10	0.69	0.09–5.42	0.72	0.53^Ŧ^	0.05–5.41	0.59
>11	1.83	0.68–4.89	0.23	1.10^Ŧ^	0.29–4.16	0.89
Respiratory insufficiency						
Post-protocol	1.23	0.77–1.97	0.38	1.05^†^	0.59–1.87	0.86
Crystalloid Bolus Volume (ml/kg)						
None	1	-	-	1	-	-
1–20	4.33	1.93–9.70	<0.001	2.07^Ŧ^	0.69–6.22	0.20
21–60	5.36	2.94–9.75	<0.001	1.96^Ŧ^	0.74–5.18	0.17
>60	6.70	2.97–15.10	<0.001	2.20^Ŧ^	0.75–6.47	0.15
Whole Blood Volume (ml/kg)						
None	1	-	-	1	-	-
1–10	12.26	1.62–92.82	0.02	4.66^Ŧ^	0.55–39.88	0.16
>11	5.64	2.16–14.69	<0.001	1.79^Ŧ^	0.50–6.40	0.37
Median length of stay >96 hours						
Post-protocol	1.59	1.03–2.47	0.04	1.81^†^	1.10–2.96	0.02
Crystalloid Bolus Volume (ml/kg)						
None	1	-	-	1	-	-
1–20	0.76	0.370–1.57	0.46	0.47^Ŧ^	0.17–1.29	0.14
21–60	1.11	0.65–1.88	0.70	0.62^Ŧ^	0.26–1.50	0.29
>60	2.40	1.21–4.75	0.01	1.53^Ŧ^	0.58–4.01	0.39
Whole Blood Volume (ml/kg)						
None	1	-	-	1	-	-
1–10	2.00	0.80–5.00	0.14	1.82^Ŧ^	0.61–5.46	0.28
>11	2.08	1.12–3.86	0.02	1.03^Ŧ^	0.46–2.32	0.94

^†^ Adjusted for age in months, male sex, WAZ, PIM2 score, hematocrit, meets sepsis criteria, culture positive sepsis

^Ŧ^ Adjusted for age in months, male sex, WAZ, PIM2 score, hematocrit, meets sepsis criteria, culture positive sepsis, antibiotics within 60 minutes, post-protocol, whole blood volume received (ml/kg), crystalloid bolus volume received (ml/kg)

The odds of fluid overload were significantly higher post-protocol (AOR of 3.45, 95% CI 2.04–5.85), as were the odds of heart failure (AOR 4.52, 95% CI, 1.43–14.29), and the odds of a longer (>96 hours) median length of stay (AOR 1.81, 95% CI 1.10–2.96). The odds of respiratory insufficiency were not statistically significantly (AOR 1.05, 95% CI, 0.59–1.87). We then stratified each outcome by type and total volume of fluid received–crystalloid bolus volume and whole blood volume (Table 4). The adjusted odds of mortality were not statistically significant for any volume of crystalloid or whole blood when compared to subjects that received no crystalloid or blood, respectively. The odds of fluid overload were decreased, though not statistically significant, for all volumes of crystalloid (AOR 0.74, 95% CI 0.26–2.12; AOR 0.78, 95% CI 0.30–1.98; AOR 0.81, 95% CI 0.29–2.25, for 1–20, 21–60 and >60 ml/kg, respectively); however, subjects that received any volume of whole blood had higher odds of fluid overload, though only >11 ml/kg was statistically significant (AOR 2.61, 95% CI 0.89–7.71, AOR 5.71, 95% CI 2.19–14.90, for 1–10, >11 ml/kg, respectively). The odds of heart failure, respiratory insufficiency, and longer median length of stay were not statistically significant for any volume of crystalloid or blood.

### Protocol compliance and clinical interventions

**Fig 4:**
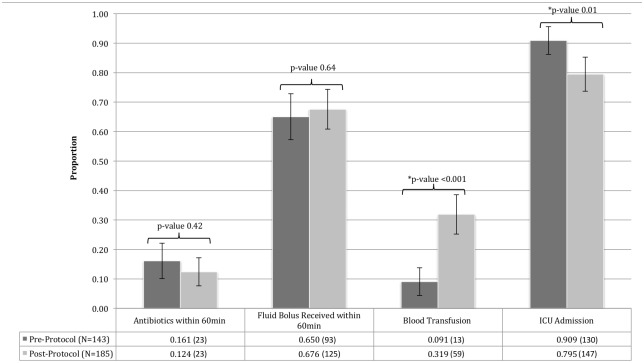
Key protocol time points and events. Bar graph shows the proportion of patients pre- and post-protocol implementation that received antibiotics within 60 minutes, the first fluid bolus received within 60 minutes, a blood transfusion, and an admission to the ICU. Error bars represent 95% confidence intervals and p-values for each comparison pre- and post-protocol are shown.

We measured protocol compliance by administration of antibiotics and the first fluid bolus within one hour of admission (Fig 4). Rates of early antibiotic and bolus administration were similar pre- and post-protocol and neither was statistically significant (16.1% vs. 12.4%, p = 0.42; 65.0% vs. 67.6%, p = 0.64, respectively). The pre-protocol cohort had a significantly higher rate of admission to the ICU (90.9% vs. 79.5%, p = 0.01), and a lower rate of blood transfusions compared to the post-protocol cohort (9.1% and 31.9%, p<0.001, respectively) (Fig 4).

We also analyzed the volume of fluid received pre- and post-protocol (Table 5) and found that the median total volume received was similar and not statistically significant (20.8 vs. 30.5 ml/kg, p = 0.16, pre- and post-protocol, respectively). Median fluid bolus and blood volumes were also similar between cohorts (20.3 vs. 24.8 ml/kg, p = 0.54, and 0 vs. 0 ml/kg, p<0.001, pre- and post-protocol, respectively), though the interquartile range (IQR) for blood volume received was greater in the post-procotol cohort.

**Table 5:** Median volume of fluid received pre- and post-protocol by fluid type (crystalloid vs. blood).

Volume received	Pre-protocol (N = 137)^†^	Post-protocol (N = 185)	p-value
Total volume (crystalloid + blood), median (IQR) (ml/kg)	20.8 (0–54.1)	30.5 (0–61.3)	0.16
Crystalloid bolus, median (IQR) (ml/kg)	20.3 (0–50)	24.8 (0–50)	0.54
Whole blood, median (IQR) (ml/kg)	0 (0–0)	0 (0–10)	<0.001

^†^ Limited to subjects with available bolus and blood volume data.

IQR: Interquartile range.

## Discussion

An evidenced-based pediatric sepsis protocol developed for resource-rich settings did not improve mortality and was associated with increased rates of fluid overload, heart failure, and median length of hospital stay in this limited resource setting. The slightly higher PIM2 score and increased percentage of children with septic shock in the post-protocol cohort suggest that this cohort had an increase in illness severity. Though mortality rates were similar in each cohort, mortality could have been higher in the absence of the Pediatric Sepsis Protocol if the severity of illness was higher in the post-protocol cohort. In the adjusted model of mortality taking into account severity of illness, we did not find a mortality benefit post-protocol implementation.

Overall, pediatric sepsis management appears to be similar before and after protocol implementation with one exception. We found that there was an increased number of blood transfusions post-protocol implementation, despite no significant clinical difference in baseline hematocrit between the two cohorts. There was not an increase in early antibiotic or fluid bolus administration post-protocol implementation, suggesting that the protocol was either not being followed or the treatment targets were not realistic for this setting.

It is possible that with implementation of the sepsis protocol, provider awareness increased and children were more quickly and accurately diagnosed and treated. Likewise, with increased sepsis awareness, there may have been a diagnosis bias post-protocol with more children diagnosed with sepsis potentially not meeting sepsis criteria. However, a member of the research team performed a chart review for all included patients and, based on documented vital signs, laboratory results, and vasoactive infusions, retrospectively determined if the patient met sepsis, severe sepsis, or septic shock criteria, as shown in Table 1. As this is a retrospective chart review and patients were identified based on a diagnosis of sepsis, it is unclear how many children met sepsis criteria but were not identified as such. Therefore, with this study alone we cannot answer the question of how well sepsis is recognized at Dhaka Hospital.

Per clinical practice at Dhaka Hospital and WHO guidelines,[21] whole blood transfusion in the ICU is indicated for patients with fluid-refractory septic shock, or in non-severely malnourished children with sclerema (Box 1). Blood transfusion is indicated for patients on the ward with severe pallor (hemoglobin <5gm/dl). More children met criteria for septic shock in the post-protocol cohort, which may explain the differential transfusion practices between the cohorts. The post-protocol cohort was not only more likely to receive blood, but also more likely to receive a higher volume of blood as compared to the pre-protocol cohort. The post-protocol subjects that died received a greater volume of blood than their pre-protocol counterparts, but with a multivariable logistic regression model, we did not find a higher odds of mortality associated with blood volume. However, the increased volume of whole blood transfusions post-protocol implementation was associated with a statistically significant increase in odds of fluid overload. This may be due to the absolute volume that a patient received (crystalloid plus blood), though this is not supported by the results of our analyses. One proposed mechanism for whole blood causing or worsening fluid overload is that exposure to highly immunogenic whole blood triggers further inflammation, compounding the ongoing systemic inflammatory process, exacerbating third spacing, and resulting in further fluid overload while increasing hospital length of stay. There is good evidence that fluid overload is associated with worse outcomes in critically ill children,[38–40] though there is currently mixed evidence in the literature regarding the potential risks and benefits of blood transfusion for patients with sepsis.[41–44] Despite the potential association between whole blood transfusion and fluid overload, we did not find an increased rate of mortality or respiratory-insufficiency in the post-protocol cohort.

Early goal-directed therapy for the management of sepsis has recently been called into question in the adult literature,[45–47] and our results, specifically surrounding aggressive fluid resuscitation and blood transfusion practices, also raise some concern. Subjects in this cohort had progressively higher adjusted odds for fluid overload with increased whole blood volume, even after controlling for malnutrition. Though the adjusted odds of mortality were similar and not statistically significant across all fluid and volume strata, the adjusted odds for median length of stay were increased in the post-protocol cohort. Especially when resources are limited, resource utilization represented by hospital length of stay, becomes a critical outcome measure. Though this study was not designed to test the effectiveness of early goal-directed therapy, our results invite interesting questions that need to be further investigated.

Pediatric sepsis protocols have been shown to be effective in resource-rich settings[23–29]. Our results raise the question of whether a pediatric sepsis protocol is effective in this limited resource setting. For example, the icddr,b Pediatric Sepsis Protocol included clinical targets, such as central venous pressure (CVP) and urine output goals, though central lines and Foley catheters are not standard of care due to a lack of resources at Dhaka Hospital. It is possible that the protocol is effective in this limited resource setting, but implementation was inadequate and/or protocol compliance was poor; our measure of protocol compliance–antibiotic and fluid bolus administration within one hour–could support either. This study cannot distinguish between the above questions, and further research is necessary.

The two cohorts were comparable across most baseline patient characteristics, with the exception of the median predicted probability of death based on the PIM2 score and the percentage of subjects with septic shock. Though there were statistically significant differences, a predicted probability of death of 2.8% compared to 3.7% is unlikely to be clinically significant, and the adjusted odds ratios did not change significantly after taking into account this difference. The PIM2 score is dependent on: laboratory data (P_a_O_2_, base excess) often not available at the time of admission to the ICU in limited resource settings; vital signs that are not routinely measured (systolic blood pressure infrequently measured in children at Dhaka Hospital); and admission diagnoses more common in resource-rich settings while neglecting frequent diagnoses in limited resource settings such as malaria, pneumonia, and malnutrition. When calculating the PIM2 score, missing data is noted as a zero, which decreases the overall score. The cumulative effect of these factors is that the PIM2 score likely grossly underestimates the illness severity in this patient population and is not an accurate assessment. Although the PIM2 score is not an ideal option to evaluate absolute severity of illness in the context of limited resource settings, it allowed for an objective comparison of severity of illness between the pre- and post-cohorts.

We determined sepsis severity by chart review, and were limited to whether vital signs, laboratory results, and vasoactive infusions were documented. It is up to the clinician to determine cardiovascular dysfunction and initiate vasoactive infusions for septic shock, as defined in Box 1. Initiation of vasoactive support is a step in the Pediatric Sepsis Protocol; it is possible that implementation of the protocol improved provider recognition of shock and initiation of vasoactive infusions, which may explain the increase in septic shock cases observed post-protocol.

It is worth mentioning that the post-protocol cohort had a slightly higher, though not statistically significant, percentage of severely malnourished children. To account for the fact that malnourished children are typically admitted to the Nutritional Rehabilitation Unit following medical stabilization and thus have a longer length of stay than their better-nourished counterparts, length of stay was adjusted for malnutrition (WAZ). Even once adjusted for possible confounding factors, the post-protocol cohort did not experience a decrease in odds of mortality and appears to have a longer median length of stay.

Pre-protocol implementation, severely malnourished children with severe sepsis received a fluid bolus over 60 minutes. With protocol implementation, the fluid bolus duration was shortened to 10–30 minutes, the same as children without severe malnutrition. Though our analyses did not show an association between fluid bolus volume and fluid overload, we did not capture data for fluid bolus duration. This change in bolus duration could certainly have contributed to the increase in fluid overload observed post-protocol, especially given the small sample size. Given that two-thirds of patients with sepsis also have severe malnutrition, Dhaka Hospital has since revised the sepsis protocol to extend the fluid bolus duration to 60 minutes in severely malnourished children and 10–30 minutes in well-nourished children.

There are several limitations to this study. The first is that this is a retrospective cohort study, and as such, we are limited by the data in the EMR. Take the measurement of time to antibiotics, for example. It is possible that physicians are immediately treating patients, especially those with severe sepsis, and documentation is not entered into SHEBA until after initial resuscitation, which often exceeds one hour. However, we do not expect this potential error to be different between the pre- and post-protocol cohorts, so it should not favor one cohort over the other. This issue of delay in reporting in SHEBA has recently been discussed in depth at Dhaka Hospital.

We were also limited by a lack of diagnostic resources and intensive monitoring that would aid in determining a definitive diagnosis and monitoring treatment effects. Similar to many limited resource settings around the world, clinicians at Dhaka Hospital, and thus this study, rely on clinical criteria and the physical exam, e.g. rales, edema, to make diagnoses, e.g. heart failure or fluid overload. There is a risk of both ascertainment bias and misclassification in determining the clinical outcomes; however, this risk is the same between the two cohorts and does not favor one cohort over the other.

Another limitation is that Dhaka Hospital specializes in the diagnosis and treatment of diarrheal disease. Though Dhaka Hospital does not turn patients away, compared to a general pediatrics hospital in urban Bangladesh, we expect Dhaka Hospital cares for more cases of diarrhea, dehydration and malnutrition. This may make the results less generalizable; however, sepsis is a widespread, global problem. We suspect the key difference between our study population and the general pediatric population in limited resource settings is primarily the source of sepsis; the pediatric population at Dhaka Hospital is more likely to have an enteric source and the general population is more likely to have parasitemia (malaria) and/or a respiratory source [12, 48].

The final limitation of this study is power. We had a restricted sample size in order to avoid confounding factors related to ICU upgrades and staff training, and although we did not detect a statistically significant difference in mortality between the two groups, our sample size was inadequate to detect any decrease ≤14%. Even a decrease in mortality of 5% would be clinically relevant, but would have required a sample size of over 2,000 children. However, given the overall similarities between pre- and post-protocol interventions and poor overall protocol compliance, we feel that it is unlikely that we would find a significant survival benefit with this protocol even with a larger sample size.

A pediatric sepsis protocol in this limited resource setting did not improve all-cause mortality. This may be due to an increased rate and volume of whole blood transfusions. Sepsis protocols may be effective in a highly controlled and monitored situation, such as a clinical trial, but perhaps the true challenge is in affecting a sustainable behavioral change among providers. Furthermore, fluid resuscitation and blood transfusion guidelines developed in resource-rich settings may not be the solution for limited resource settings. A pediatric sepsis protocol must take into account the local context; the unique patient population and its co-morbidities, co-infections, and etiologies of sepsis; and available resources including personnel. Based on the results of this study, future efforts and studies should focus on: potential barriers to the diagnosis and treatment of pediatric sepsis in limited resource settings; the development of a sepsis protocol designed specifically for limited resource settings; successful implementation strategies including educational interventions for all members of the treatment team with a focus on enduring human behavioral change; and the efficacy of sepsis protocols tailored to available resources and the specific population.

## Supporting information

S1 Table Pediatric Index of Mortality 2 Score (PIM2)[37]. (DOCX)
