# Supplementary material for: Clinical outcomes and mortality before and after implementation of a pediatric sepsis protocol in a limited resource setting: A retrospective cohort study in Bangladesh
Source: PLoS One. 2017 Jul 28;12(7):e0181160. doi: 10.1371/journal.pone.0181160 (PMC5533322; doi:10.1371/journal.pone.0181160)
Supplement: S1 Table — (DOCX) [file pone.0181160.s001.docx]

| ICU Admission Finding* | Coefficient | Coding rules |
| --- | --- | --- |
| Absolute systolic blood pressure, mmHg | 0.01395 | Unknown = 120 |
| Pupils fixed to light | 3.0791 | >3 mm and both fixed = 1  Other or unknown = 0 |
| 100X F_i_O_2_/P_a_O_2_, mmHg^-1^ | 0.2888 | Unknown =0 |
| Absolute base excess in arterial or capillary blood, mmol/l | 0.1040 | Unknown =0 |
| Mechanical ventilation at any time during the first hour in ICU | 1.3352 | Yes = 1  No = 0 |
| Elective admission | -0.9282 | Yes = 1  No = 0 |
| Recovery from surgery or procedure is main reason for admission | -1.0244 | Yes = 1  No = 0 |
| Admitted following cardiac bypass | 0.7507 | Yes = 1  No = 0 |
| High risk diagnosis (yes/no) | 1.6829 | None or if in doubt = 0 Cardiac arrest preceding admission = 1 Severe combined immune deficiency = 2 Leukemia or lymphoma after first induction = 3 Spontaneous cerebral hemorrhage = 4 Cardiomyopathy or myocarditis = 5 Hypoplastic left heart syndrome = 6 HIV infection = 7 Liver failure main reason for admission = 8 Neuro-degenerative disorder = 9 |
| Low risk diagnosis (yes/no) | -1.5770 | None or if in doubt = 0  Asthma main reason for admission = 1 Bronchiolitis main reason for admission = 2 Croup main reason for admission = 3 Obstructive sleep apnea main reason for admission = 4 Diabetic ketoacidosis main reason for admission = 5 |
| Constant = -4.8841 | | |

**Supplementary 1 Table. Pediatric Index of Mortality 2 Score (PIM2)(** [**37**](#_ENREF_37)**)**

* First value of each variable measured from the time of first contact to 1 hour after arrival to the ICU

Logit = (-4.8841) + (values * β) + (0.01395 * (absolute (SBP-120))) + (0.1040 * (absolute base excess)) + (0.2888 * (100*F_i_O_2_/P_a_O_2_))
Predicted death rate = e^Logit^/ (1+e^Logit^)
